# The Future of Vaccine Therapy

**Published:** 1919-04-12

**Authors:** 


### THE FUTURE OF VACCINE THERAPY.

Since the publication of details of the thorough and technically beautiful research work by Sir Almroth Wright and his colleagues there have arisen a number of attempts to explain to the public the frequent and unexpected failures of vaccine treatment in the past. The authors have undoubtedly put forward clear and experimentally well-supported theories for these disappointing variations, but the real value of this work lies more in the lines of progress it both makes and suggests, than in any retrospective comments. Naturally the clearing of obscure points will be invaluable to the clinician in guidance of his choice of conditions under which inoculation may be expected to succeed, and one may be excused at the outset for giving illustration of two such applications.

First, in the instance of continuous or recurrent inflammation and pyrexia, with bacterial foci pouring toxins into the circulating body-fluids so frequently that every cell in the system is doing its utmost and yet cannot get the upper hand, it is useless to give a provocative vaccine injection. The reserves of the body are already mobilised, and the call for more must inevitably be in vain. Secondly, taking the case of organisms localised and already surrounded by a zone in which their poisons are predominant, all the forces, cellular or fluid, which an inoculation may stimulate to attack, will be repelled with relative ease by this surrounding defence, an *ec-phylactic* area. The larger the area, the more secure the germs, and one sees the process exemplified in the success of vaccine treatment for furunculosis with multiple small lesions and the failure with almost any large enclosed focus.

One gives this brief outline in view of the fact that, a few years ago, popular imagination was so held by the possibilities of the then new injection treatments that, in practice, a large amount of relatively haphazard work was done, and this often in obscure cases which fell into either class mentioned above but showed no signs sufficiently definite to call for rational treatment of rest or immobilisation in the first type and surgical evacuation in the second. As a result, there is to-day a tendency to doubt and perhaps unduly to criticise the bacteriological therapy, and it is with the object of dispelling such ideas that the details and value of Sir Almroth Wright's work should be brought prominently before the profession and public alike. To the former, details of immense interest should be available at the clinical meeting of the British Medical Association now being held at South Kensington, and one is glad to see that the daily papers have already considered the matter worthy of public attention.

In the press, however, there does not seem to be sufficient stress laid upon two most important advances, (*a*), the discovery that inoculation with the *specific* germ is not always essential to success; failure does not mean that further and further search for the exact causal organism is the only efficient method. Provocative inoculation has been shown to raise the anti-power of the body to organisms other than those actually injected, *e.g.*, men inoculated against typhoid are found to be considerably protect against "other diseases," even malaria; and (*b*), the fact is now established that blood drawn from a normal patient can be artificially immunised *in vitro* by the addition of a vaccine under laboratory conditions. The number of organisms can be varied and that giving the highest immune power determined without the presence of the patient. This obviates, in the first place, speculative first injections into a sick man, and in the second, allows the direct transfusion of highly bactericidal fluid into a human system the utmost resources of which have been already called upon, that is, provides direct treatment for the first class of case we described as an almost invariable failure with the older methods.

All can read with both advantage and pleasure Sir Almroth's most understandable writings, and if his recent advances fulfil their early promise, vaccine therapy, instead of being a partial failure in all except preventive inoculation, may yet become one of the most valuable, if not the absolute, means of combating any microbic infection, whether newly implanted or in the chronic stage.

